# Rituximab as a First-line Treatment for Autoimmune Hemolytic Anemia in Multicentric Castleman's Disease

**DOI:** 10.7759/cureus.59080

**Published:** 2024-04-26

**Authors:** Jose L Cáceres Medina, Luis A González Torres, Alan Gamboa-Meza, Olga G Cantu-Rodriguez

**Affiliations:** 1 Internal Medicine, Hospital Universitario Dr. José Eleuterio González, Universidad Autónoma de Nuevo León, Monterrey, MEX; 2 Pulmonary and Critical Care Medicine/Internal Medicine, Hospital Universitario Dr. José Eleuterio González, Universidad Autónoma de Nuevo León, Monterrey, MEX; 3 Hematology, Hospital Universitario Dr. José Eleuterio González, Universidad Autónoma de Nuevo León, Monterrey, MEX

**Keywords:** coombs test, lymphoproliferative disorder, symptomatic anemia, idiopathic multicentric castleman's disease, autoimmune hemolytic anemia (aiha)

## Abstract

Castleman´s disease (CD) is a rare lymphoproliferative disorder. Concurrent autoimmune disease and CD are uncommon, but even more so, comorbid CD and autoimmune hemolytic anemia (AIHA). To the best of our knowledge, this case represents the first successful AIHA and multicentric CD (MCD) treatment using rituximab as first-line treatment. We present the case of a 53-year-old woman with a 10-year history of plasma cell variant CD who arrived at the emergency department with signs and symptoms of anemia. On admission, we made a preliminary diagnosis of hemolytic anemia and initiated immunosuppressive therapy with rituximab and steroids. After seven days, the patient recovered according to clinical and laboratory parameters, and we discharged her early. We portray a rare occurrence of CD and AIHA successfully treated with rituximab and steroid therapy, which makes our case unique.

## Introduction

Castleman disease (CD) is a rare lymphoproliferative disorder classified into two types: unicentric CD (UCD) and multicentric CD (MCD). There are three histologic subtypes: hyaline vascular (80%), plasma cell (10%), and mixed variants [1]. Autoinflammatory mechanisms may play an essential role in its development, as evidenced by a systematic review of 1923 cases reported in 38 patients with positive autoantibodies or autoimmune hemolytic anemia [2,3]. A few cases describe simultaneous autoimmune hemolytic anemia (AIHA) and CD occurrence.

Our case portrays this rare clinical scenario with the added value of treatment experience using rituximab and steroids. Two other case reports have reported the direct intention of using rituximab as a treatment, of which one was successful. This case describes the first successful treatment of AIHA and MCD using rituximab as first-line treatment.

## Case presentation

A 53-year-old woman with a 10-year history of "plasma cell variant CD" presented to the Emergency Department (ED) with a three-month history of fatigue, moderate effort-dependent dyspnea, and new-onset jaundice the day before the presentation.

**Figure 1:**
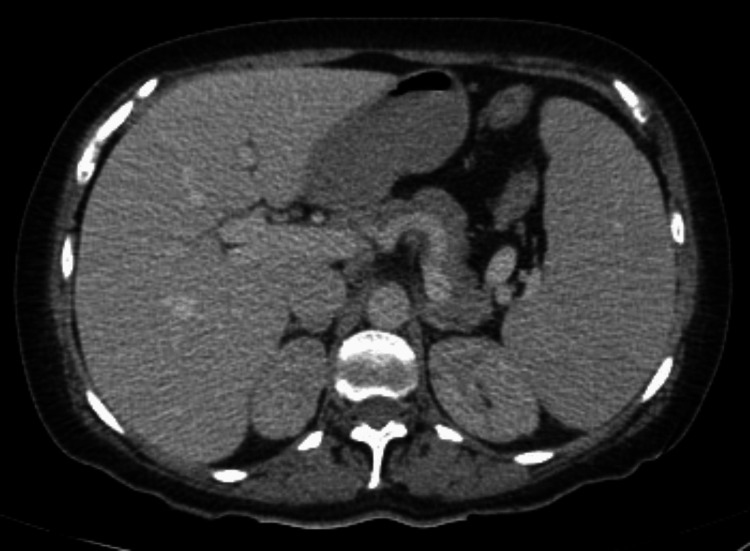
Contrast-enhanced abdominal CT scan in venous phase demonstrating hepatosplenomegaly, enlarged liver (21.4 x 17.5 cm) and spleen (17.1 x 15.2 cm)

**Figure 2:**
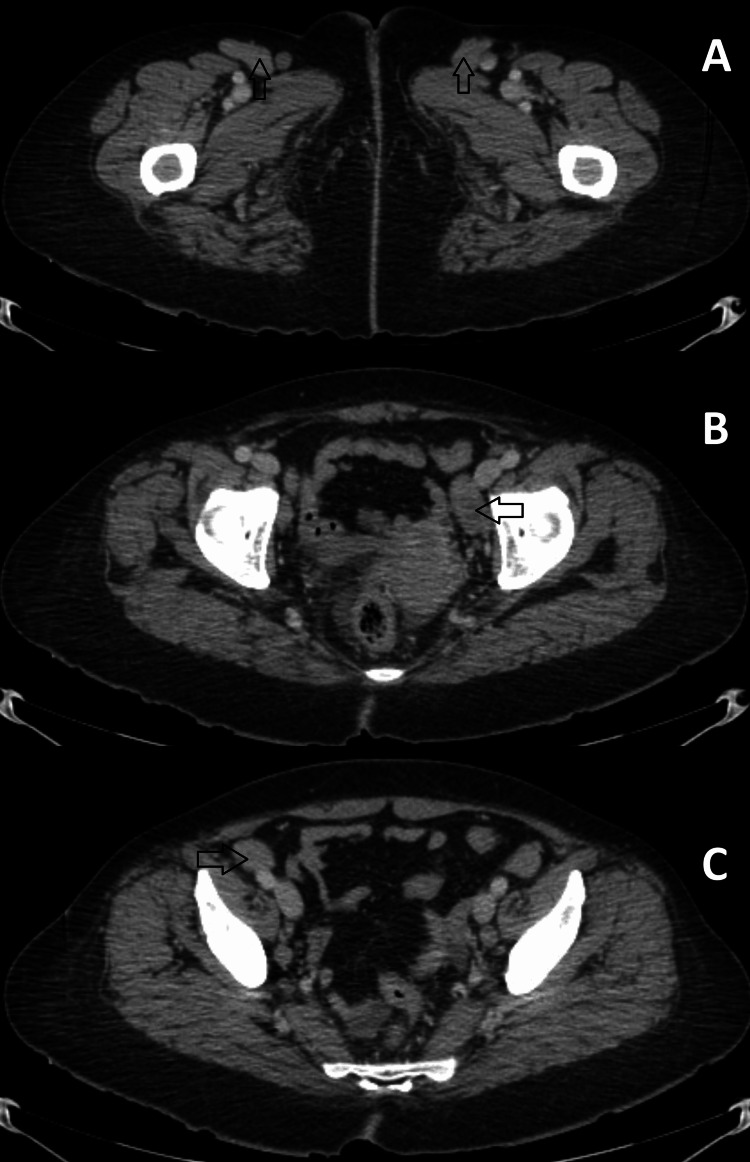
Contrast-enhanced abdominal CT scan in venous phase showing multiple adenopathies (A) Right (3.8 x 1.3 cm) and left (2.9 x 1.4 cm) inguinal adenopathies (arrows); (B) Left external iliac adenopathy (2.9x 1.4 cm) (arrow); (C) Right external iliac adenopathy (2.6 x 1.8 cm) (arrow).

On admission, her vital signs showed tachycardia with no other abnormalities. The physical examination revealed generalized jaundice, hepatosplenomegaly, and bilateral superficial inguinal adenopathies. The laboratory results showed regenerative macrocytic anemia (hemoglobin: 3.4 g/dL, mean corpuscular volume: 110 fL, reticulocytes: 45%), elevated lactate dehydrogenase (LDH), indirect hyperbilirubinemia (total bilirubin: 5.0 mg/dL, direct bilirubin: 0.3 mg/dL, indirect bilirubin: 4.7 mg/dL, LDH: 376 U/L), and normal liver enzymes. We performed a Coombs test, which yielded a triple cross-positive result. Subsequently, we conducted a monospecific IgG test, which confirmed warm antibody AIHA. Hematology recommended administering dexamethasone at 40 mg every 24 hours for four days, followed by a daily dosage of 75 mg of prednisone. Rituximab was advised at a dose of 600 mg weekly for four weeks, along with a CD assessment. An abdominal computed tomography (CT) was performed, revealing hepatosplenomegaly and bilateral inguinal and external iliac lymph nodes (Figure 1 and Figure 2).

After seven days, the patient recovered substantially and exhibited no AIHA activity. By the third and 12th-month follow-up visits, we had detected no CD or AIHA activity.

## Discussion

We presented a case of comorbid CD and AIHA treated with rituximab as a first-line therapy. To the best of our knowledge, this case represents the first successful AIHA-MCD treatment using rituximab and should serve as a guide when encountering patients with similar clinical characteristics. Our main challenge was the inability to attribute the complete response to rituximab therapy solely. Additionally, the lack of histopathological imaging for the excisional biopsy of adenopathies posed another limitation in our case.

Anemic disorders are a frequent feature of the plasma cell variant of CD, but hemolytic anemia is not a common finding in CD [3]. Arthritis, proteinuria, Sicca syndrome, polyneuropathy (without diagnosing POEMS (Polyneuropathy, Organomegaly, Endocrinopathy, Monoclonal gammopathy, and Skin abnormalities) syndrome), interstitial lung disease, immune thrombocytopenic purpura, and AIHA are all autoimmune characteristics of MCD. Comorbid autoimmune diseases in CD include myasthenia gravis, psoriasis, amyloidosis, sarcoidosis, and others [4]. Liu et al. discovered that among 1923 CD cases, 38 exhibited autoantibodies indicative of AIHA [2]. Another large series of patients with UCD reported AIHA in only one out of 71 patients [5].

The literature shows more than 10 cases of AIHA with CD and four cases of Evans syndrome with CD. Treatment consistently involves steroids, with varying use of tocilizumab, rituximab, or chemotherapy. In a few reported persistent cases, splenectomy was the definitive treatment. Our patient presented with MCD and AIHA, and we decided to treat her with steroids and rituximab, to which she had a prolonged response. Monoclonal therapies appear to be therapeutical options when considering autoimmune coexistence with MCD. Table 1 summarizes monoclonal antibody use in CD presenting with AIHA [6-8].

**Table 1:** Reported cases of monoclonal antibody therapy for CD patients presenting with AIHA CD: Castleman´s disease; MCD: multicentric Castleman's disease; AIHA: autoimmune hemolytic anemia; CHOP-R: cyclophosphamide, doxorubicin, vincristine, prednisolone, and rituximab

Case Report	Description
Tajima et al. [6]	A 50-year-old male with MCD + AIHA; CHOP-R success, Rituximab may have contributed to AIHA remission.
Tabata et al. [7]	A 43-year-old with MCD + AIHA; tocilizumab failure, success using rituximab.
Plano et al. [8]	A 72-year-old male with MCD + AIHA; rituximab failure, success using siltuximab.

Steroid therapy represents the initial treatment choice for AIHA. A comparative analysis from a small clinical trial examined the effectiveness of prednisolone alone versus a combination with rituximab therapy. The trial reported a success rate of 36% with prednisolone alone, contrasting significantly with a success rate of 75% when combined with rituximab therapy within 12 months [9].

A study by Barcellini et al., encompassing 308 cases of AIHA, pointed out that the emergence of severe anemia at disease onset is associated with elevated hazard ratios for relapse and an increased requirement for therapeutic interventions [10]. Additionally, their evaluation reported a 14% increase in relapse risk for every gram reduction of hemoglobin.

Given the rarity of similar cases, MCD activity proof, and the presentation of severe anemia, we opted to initiate second-line treatment as the primary approach. We observed a good response after 12 months.

## Conclusions

We encountered a clinical scenario where a well-known disease met with a rarer one. Physicians should consider a diagnosis of AIHA in patients with a history of MCD, severe anemia, and constitutional signs. Rituximab is a viable first-line option for treating AIHA related to MCD. Little evidence supports monoclonal body use in AIHA and active MCD, and this case report presents the first successful experience of rituximab as a first-line treatment. Further knowledge, case reporting, and evidence review are needed when considering concomitant MCD with AIHA.

## References

[1] Rodrigo C, Rajapakse S, Gooneratne L (2015). Rituximab in the treatment of autoimmune haemolytic anaemia. Br J Clin Pharmacol.

[2] Liu AY, Nabel CS, Finkelman BS (2016). Idiopathic multicentric Castleman’s disease: a systematic literature review. Lancet Haematol.

[3] Fajgenbaum DC (2018). Novel insights and therapeutic approaches in idiopathic multicentric Castleman disease. Blood.

[4] González García A, Fernández-Martín J, Robles Marhuenda Á (2023). Idiopathic multicentric Castleman disease and associated autoimmune and autoinflammatory conditions: practical guidance for diagnosis. Rheumatology (Oxford).

[5] Boutboul D, Fadlallah J, Chawki S (2019). Treatment and outcome of unicentric Castleman disease: a retrospective analysis of 71 cases. Br J Haematol.

[6] Tajima K, Yamamoto H, Suzuki I (2013). Autoimmune hemolytic anemia with warm-reactive immunoglobulin M antibody in multicentric Castleman disease. Ann Hematol.

[7] Tabata S, Higuchi T, Tatsukawa S, Narimatsu K, Takeo H, Matsukuma S, Ito T (2019). Idiopathic multicentric Castleman disease with autoimmune hemolytic anemia and production of anti-drug antibody against tocilizumab. Intern Med.

[8] Plano F, Mancuso S, Camarda GM (2024). A multicentric Castleman disease associated with mixed warm and cold antibody-mediated aha responsive to siltuximab. Chemotherapy.

[9] Birgens H, Frederiksen H, Hasselbalch HC (2013). A phase III randomized trial comparing glucocorticoid monotherapy versus glucocorticoid and rituximab in patients with autoimmune haemolytic anaemia. Br J Haematol.

[10] Barcellini W, Fattizzo B, Zaninoni A (2014). Clinical heterogeneity and predictors of outcome in primary autoimmune hemolytic anemia: a GIMEMA study of 308 patients. Blood.

